# Revealing metastatic castration‐resistant prostate cancer master regulator through lncRNAs‐centered regulatory network

**DOI:** 10.1002/cam4.6481

**Published:** 2023-08-29

**Authors:** Rafaella Sousa Ferraz, João Vitor Ferreira Cavalcante, Leandro Magalhães, Ândrea Ribeiro‐dos‐Santos, Rodrigo Juliani Siqueira Dalmolin

**Affiliations:** ^1^ Laboratory of Human and Medical Genetics, Institute of Biological Sciences Federal University of Para Belem Brazil; ^2^ Bioinformatics Multidisciplinary Environment—IMD Federal University of Rio Grande do Norte Natal Brazil

**Keywords:** long noncoding RNA, mCRPC, system biology, transcriptional network reconstruction

## Abstract

**Background:**

Metastatic castration‐resistant prostate cancer (mCRPC) is an aggressive form of cancer unresponsive to androgen deprivation therapy (ADT) that spreads quickly to other organs. Despite reduced androgen levels after ADT, mCRPC development and lethality continues to be conducted by the androgen receptor (AR) axis. The maintenance of AR signaling in mCRPC is a result of AR alterations, androgen intratumoral production, and the action of regulatory elements, such as noncoding RNAs (ncRNAs). ncRNAs are key elements in cancer signaling, acting in tumor growth, metabolic reprogramming, and tumor progression. In prostate cancer (PCa), the ncRNAs have been reported to be associated with AR expression, PCa proliferation, and castration resistance. In this study, we aimed to reconstruct the lncRNA‐centered regulatory network of mCRPC and identify the lncRNAs which act as master regulators (MRs).

**Methods:**

We used publicly available RNA‐sequencing to infer the regulatory network of lncRNAs in mCRPC. Five gene signatures were employed to conduct the master regulator analysis. Inferred MRs were then subjected to functional enrichment and symbolic regression modeling. The latter approach was applied to identify the lncRNAs with greater predictive capacity and potential as a biomarker in mCRPC.

**Results:**

We identified 31 lncRNAs involved in cellular proliferation, tumor metabolism, and invasion‐metastasis cascade. SNHG18 and HELLPAR were the highlights of our results. SNHG18 was downregulated in mCRPC and enriched to metastasis signatures. It accurately distinguished both mCRPC and primary CRPC from normal tissue and was associated with epithelial–mesenchymal transition (EMT) and cell‐matrix adhesion pathways. HELLPAR consistently distinguished mCRPC from primary CRPC and normal tissue using only its expression.

**Conclusion:**

Our results contribute to understanding the regulatory behavior of lncRNAs in mCRPC and indicate SNHG18 and HELLPAR as master regulators and potential new diagnostic targets in this tumor.

## 1 INTRODUCTION

Prostate cancer (PCa) is the second most prevalent type of cancer and the fifth leading cause of cancer‐related deaths in men worldwide.^1^ The androgen deprivation therapy (ADT) is considered the standard of care for men with high‐risk localized or advanced PCa.^2^ After 2–3 years of ADT, some patients become refractory to ADT and progress to a more aggressive form termed castration‐resistant prostate cancer (CRPC) that continues to grow even at low androgen levels^3^, ^4^, ^5^ and commonly advance to incurable and deadly metastatic CRPC (mCRPC).^6^ The goal of mCRPC therapies is to enhance patient survival and their overall quality of life. The lack of an optimal treatment strategy for mCRPC primarily stems from its significant molecular heterogeneity.^7^ Moreover, the therapeutic sequence depends on the patient's overall health, presence of comorbidities, ability to tolerate side effects, and efficacy of previous treatment.^8^, ^9^ Generally, the treatment consists of a combination of strategies, including conventional therapies (surgery, chemotherapy, and radiotherapy), hormonal therapies, immune checkpoint inhibition, and others, where these different combinations offer benefits to distinct subsets within the mCRPC population.^9^, ^10^, ^11^ Taking these factors into account, it becomes crucial to explore novel therapies to enhance clinical results, reach a larger portion of patients, and minimize avoidable side effects for individuals with mCRPC.

Despite the CRPC being unresponsive to androgens, the androgen receptor (AR) signaling axis remains the most critical factor for CRPC development, bypassing the androgen depletion through AR overexpression, de novo androgen synthesis, and AR activation induced by coregulatory proteins, mutation, or altered splicing.^4^, ^12^ In addition to AR signaling, other regulatory mechanisms have been described as involved with CRPC, including hedgehog pathway, PI3K‐AKT–mTOR signaling, Wnt pathway, and dynamic of noncoding RNAs such as long noncoding RNAs (lncRNAs).^12^ lncRNAs constitute a class of RNAs with a length of more than 200 nucleotides and rare protein‐coding potential.^13^ These molecules can act on chromosome architecture, chromatin accessibility, transcriptional machinery, splicing control, and interact with RNA‐binding proteins (RBPs) and miRNAs, affecting mRNAs transport and stability, protein translate and assembly, cytoskeleton dynamics, cell–cell interactions, and mitochondrial function.^14^ LncRNAs have been extensively studied in various physiological and pathological processes, including cancer.^15^ Given their ability to regulate biological mechanisms and its expression often restricted to specific cells, they have been proposed as potential diagnostic biomarkers and therapeutic targets.^16^

The lncRNAs participation in PCa physiopathology is not new, mainly those related to AR signaling. HOTAIR, for example, is a lncRNA upregulated in PCa that promotes proliferation and invasion in CRPC through androgen‐independent AR activation triggered by AR transcriptional stimulus and AR protein stability.^17^ Another example is PCA3, a lncRNA upregulated in more than 95% of PCa patients that modulates AR cofactors and epithelial–mesenchymal transition (EMT) markers.^18^ In 2012 the USA FDA (Food and Drug Administration) approved the diagnostic use of PCA3 to manage risk assessment of prior negative prostate biopsy.^19^ Despite the number of noncoding RNAs studies in PCa, the lncRNA regulatory mechanisms involved in the mCRPC remains unclear.

The reconstruction of transcriptional regulatory networks based on regulatory hubs is a successful approach to investigating cancer biological mechanisms and elucidating drivers of tumor behavior.^20^, ^21^, ^22^, ^23^, ^24^, ^25^ This reverse‐engineering method is based on mutual information and can identify key regulatory elements that trigger a phenotype.^26^, ^27^ This approach was previously used to build transcriptional factor‐centered regulatory networks but has recently applied to other molecules, such as lncRNAs.^28^, ^29^, ^30^ Here, we reconstructed the lncRNA regulatory network of mCRPC and identified lncRNAs acting as master regulators mediating the cell proliferation and metastatic process in mCRPC.

## 2 MATERIALS AND METHODS

### 2.1 Data acquisition and processing

**FIGURE 1:**
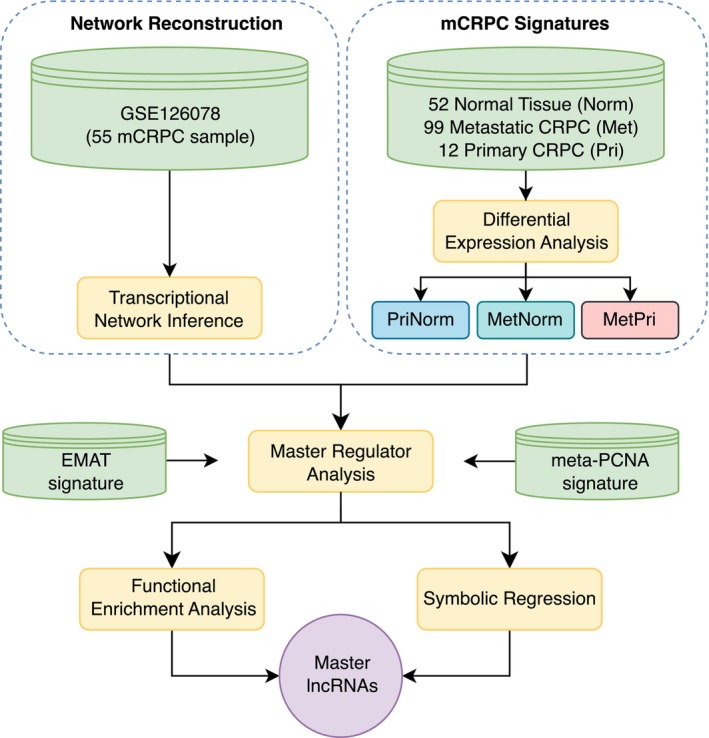
lncRNA regulatory network and master regulators inference. Expression data from GSE126078 was used to network inference, while TCGA‐PRAD (normal sample), WCDT‐MCRPC (metastatic CRPC), and GSE80609 (primary CRPC) were used to get the mCRPC signatures. The network was further analyzed by two different gene signatures: EMAT and meta‐PCNA. Inferred master regulators were then subjected to functional enrichment and symbolic regression modeling. In the end, a set of master lncRNAs that play a role in the metastatic process were identified.

Figure 1 shows the general workflow used in this study. All expression data used to infer the mCRPC network were obtained from Gene Expression Omnibus (GEO) with accession number GSE126078. This dataset comprises expression data (RNA‐Seq profiling HiSeq 2500) of 98 biospecimens of mCRPC sites obtained from 55 men who died of mCRPC. Fifty‐three of these had detected bone metastases, and 54 received androgen ablation therapy. Moreover, these patients also received other drugs, including docetaxel, abiraterone, enzalutamide, or both abiraterone and enzalutamide. To avoid sampling bias, we selected only one metastatic site from each patient, prioritizing the most frequent sites in the cohort (liver and lymph node) (Table 1).

**TABLE 1:** Frequency of tumor site used to build mCRPC network.

Tumor site	*N* (%)
Liver	29 (52.73)
Lymph node	17 (30.91)
Bone	3 (5.45)
Lung	2 (3.64)
Others^a^	4 (7.27)

^a^ Others include left posterior, pancreas, pelvic mass, and retroperitoneal tissue.

The raw RNA‐Seq data were downloaded and converted to FASTQ using SRATooKit v.3.0.0 (https://github.com/ncbi/sra‐tools). Quality control was performed using FastQC v.0.11.9^31^ and summarized with MultiQC.^32^ Fastp v.0.23.2^33^ was used to trim the adapters and filter reads with low quality. The resulting FASTQ files were mapped to GRCh38 and quantified using Salmon v.0.9.1.^34^ Gene annotation was derived from GENCODE Release 40,^35^ and transcript level abundance was inferred with tximport v.1.26.1 R package.^36^

We built three gene signatures for the master regulator analysis (MRA): (i) metastatic versus primary tumor (MetPri), (ii) metastatic versus normal tissue (MetNorm), and (iii) primary tumor versus normal tissue (PriNorm). Normal and metastatic samples were acquired from the TCGA Consortium. The TCGA‐PRAD dataset provided normal samples, while the WCDT‐MCRPC dataset provided metastatic samples. Primary CRPC samples were obtained from GEO with accession number GSE80609 and processed with the same pipeline of the TCGA. The gene signatures were built through Differential Expression Analysis using DESeq2 v.138.3 R package.^37^ Genes with FDR‐adjusted *p*‐value ≤0.01 and absolute Log_2_FoldChange ≥1.5 were considered differentially expressed and, therefore, participants of the gene signature.

Two other signatures were also used: Epithelial‐to‐mesenchymal‐to‐amoeboid transition (EMAT)‐related gene signature,^38^ which include pathways important during the metastatic cascade, and proliferative‐related gene signatures (meta‐PCNA).^39^ We verified the lncRNA expression in metastatic tissue using the same expression data used in MRA. Statistics analysis was performed by Mann–Whitney test with FDR correction, where *p*‐value ≤0.01 was considered statistically significant.

### 2.2 Transcriptional network inference and master regulator inference

Transcriptional network inference was performed using RTN v.2.22.1 R package, a tool based on the Algorithm for the Reconstruction of Accurate Cellular Networks (ARACNe) method.^26^ We selected the lncRNAs expressed in at least 90% of the metastatic samples. This filter resulted in 1770 lncRNAs that were then used to build the lncRNA‐mRNA network. The regulatory units (regulons) formed by regulatory elements (lncRNAs) and their putative targets (protein‐coding mRNAs) were computed by mutual information (MI). Permutation analysis and bootstrapping were used to remove associations below the MI threshold and unstable interactions, respectively, creating a consensus network. The data processing inequality (DPI) theorem removed redundant associations and preserved dominant regulatory‐target pairs. BH adjusted *p*‐value ≤0.01 and permutation *n* = 1000 were used as parameters in the network reconstruction. The regulatory activity was inferred by Spearman correlation between each regulatory element and its putative targets (BH adjusted *p*‐value ≤0.01). The network visualization was performed by RedeR v.2.2.1 package.^40^

We filtered the regulons with at least 15 targets for transcriptional regulatory analysis and implemented the master regulator analysis (MRA) method in the RTN package. This algorithm computes the main regulatory elements (master regulators/MRs) through statistical overrepresentation of gene signature in the regulons and points out putative lncRNAs relevant to mCRPC.

### 2.3 Functional enrichment analysis

The biological function of the regulons was investigated through the functional enrichment of the genes regulated by each MR. Enrichment analyses were conducted with the Gene Ontology Biological Process^41^ using overrepresentation analysis implemented in clusterProfiler v.4.6.0. Enriched terms with a BH adjusted *p*‐value ≤0.01 were considered statistically significant.

### 2.4 Symbolic regression

Symbolic regression (SR) is a supervised machine learning technique that tries to uncover mathematical models that fit a given dataset. This is done by searching the combinatorial space of mathematical operators and inputs for equations that predict an expected outcome with the least amount of error and complexity.^42^ In this study, we used the QLattice SR implemented in Feyn v.3.0.5 Python Package to explore the relationships between previously inferred MRs and clinical groups.^43^, ^44^ Like most SR tools, QLattice employs genetic programming to search the combinatorial space more efficiently. Briefly, it starts by generating a set of functions that prioritize features based on their mutual information with the outcome. The best performing functions are selected to make up a second generation of functions. The second generation includes mutated and combined versions of the previous best performers (by adding, removing, or swapping terms; modifying constants; etc). This process goes on over many generations so that functions converge at predicting outcomes.^43^, ^44^ For this analysis we used samples obtained from TCGA Consortium (TCGA‐PRAD for normal tissue and WCDT‐MCRPC for metastatic CRPC) and from GEO (GSE80609 for primary CRPC), that is, the same datasets used in the gene signature construction for inference of master regulators (Figure 1). We normalized the raw counts with the CPM (counts per million) method by edgeR v.3.40.2 R package,^45^ and split the data into 33% for training and 67% for testing. The best 10 models were selected by accuracy, complexity, area under curve (AUC), and Bayesian information criterion (BIC). The mathematical expressions generated are wrapped with the logistic regression function 1/(1 + exp (−*f*(*X*))) referred to in the text as logreg.

### 2.5 Competing endogenous RNAs (ceRNAs) network

ENCORI (ENCyclopedia Of RNA Interactome—https://rnasysu.com/encori/) was used to investigate the interaction between HELLPAR/SNHG18 and miRNAs. In addition, we investigated the interaction between HELLPAR/SNHG18 targets and miRNAs to evidence activity of these lncRNAs as ceRNAs (competing endogenous RNA). Only interactions observed in the prostate cancer cell line (22RV1) were maintained in the analysis. The network visualization was performed by RedeR v.2.2.1 package.^40^

## 3 RESULTS

### 3.1 mCRPC regulatory network and master regulator analysis

**FIGURE 2:**
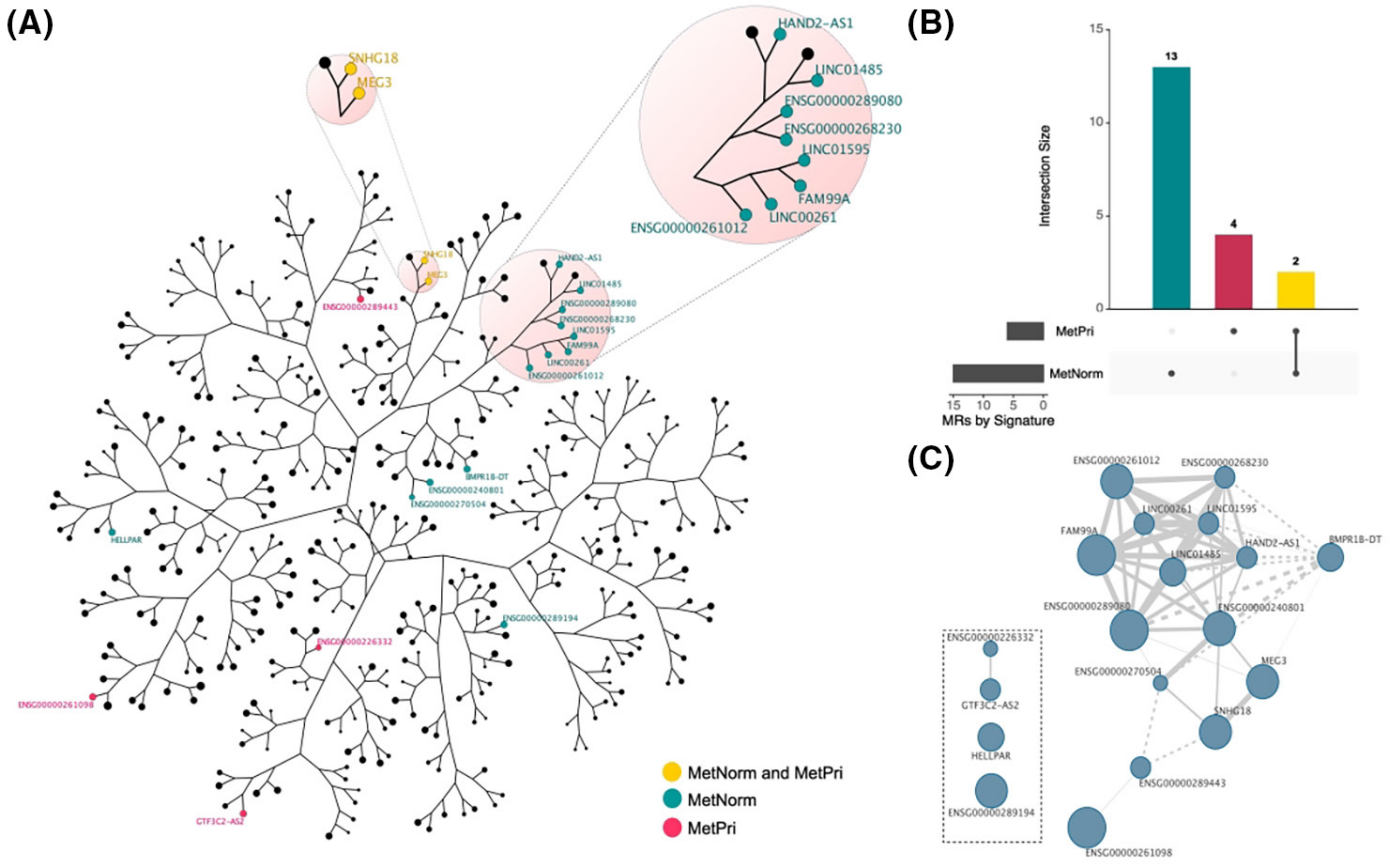
mCRPC lncRNA‐centered regulatory network. (A) Tree‐and‐leaf representation shows the mCRPC regulatory network. The nodes depict regulons labeled with lncRNA and are colored based on the master regulator analysis (MRA). The proximity of nodes in the figure corresponds to the degree of target shared between them. (B**)** Upset plot shows the number of master regulators (MRs) shared between gene signatures. (C**)** Association map shows the interaction between MRs from MetPri and MetNorm signatures. The nodes represent the regulons, and node size reflects the number of regulated genes. The edges represent the connection between regulons, and the edge width reflects the number of shared genes between a pair of regulons. Dotted edges represent antagonistic regulation, while solid edges represent agonistic regulation.

In the first step of the analysis, we reconstructed the mCRPC lncRNAs‐centered regulatory network. The analysis goal was to identify the regulons, that is, the units formed by a lncRNA and its target genes. In the next step, we performed the master regulator analysis (MRA) to identify the regulons enriched to mCRPC gene signatures called master regulators (MRs). MRs are key elements responsible for coordinating regulatory mechanisms behind mCRPC phenotype and are potential diagnostic and prognostic biomarkers. Figure 2A shows a tree‐and‐leaf representation of the mCRPC regulatory network, with nodes indicating the 375 regulons identified during reconstruction analysis, and the edges implying the overlap of these regulons hierarchically organized. Closer the nodes are in the representation, the more targets they share and, possibly, the more common biological functions they have. We used three signatures to identify mCRPC MRs: (i) metastatic versus primary tumor (MetPri), (ii) metastatic versus normal tissue (MetNorm), and (iii) primary tumor versus normal tissue (PriNorm). Overall, 10 MRs were inferred in the MetPri signature, 16 MRs in the MetNorm, and 12 MRs in the PriNorm (Figure S1).

We focused on MRs of MetNorm and MetPri signatures aiming to detect lncRNAs specifically involved in the metastatic process. These MRs are represented in Figure 2A as collared nodes, where red nodes indicate regulons enriched by MetPri signature, blue nodes indicate regulons enriched by MetNorm signature, and yellow nodes represent those enriched by both MetPri and MetNorm signature. More than half of MetNorm MRs cluster together in the network, suggesting that they share target genes and perform related regulatory functions. The two MRs shared by MetNorm and MetPri (SNHG18 and MEG3) also grouped, indicating interconnection between them (Figure 2A). The majority of the MRs are agonists, that is, regulates shared genes in the same direction (Figure 2C and Figure S2). However, some MRs demonstrate antagonistic regulation, that is, regulate shared genes in the opposite direction (Figure 2C). BMPR1B‐DT, for example, is antagonistic to every other MR it interacts with, while ENSG00000289443 was antagonistic to ENSG00000270504 and SNHG18. Some MRs regulate protein‐coding genes and other lncRNAs, including lncRNAs members of the MetNorm and MetPri signatures (Table S1).

We also identified MRs associated with two other predefined signatures related to metastatic and proliferative mechanisms, namely epithelial‐to‐mesenchymal‐to‐amoeboid transition signature (EMAT) and meta‐PCNA signature, respectively (see methods). SNHG18, ENSG00000289273, and LINC01614 were identified as MRs for EMAT signature (Figure S3). The inference of SNHG18 as MRs by all metastatic signatures used in the present study (MetNorm, MetPri, and EMAT signatures) reinforces their relevance in the mCRPC metastatic process. ENSG00000197332, ENSG00000187951, ENSG00000289194, ENSG00000260293, ENSG00000275158, and ENSG00000280206 were inferred as MRs by meta‐PCNA signature (Figure S3), suggesting that these MRs may be more associated with tumor growth than with spread of cancer.

### 3.2 Biological function inference of master regulators

**FIGURE 3:**
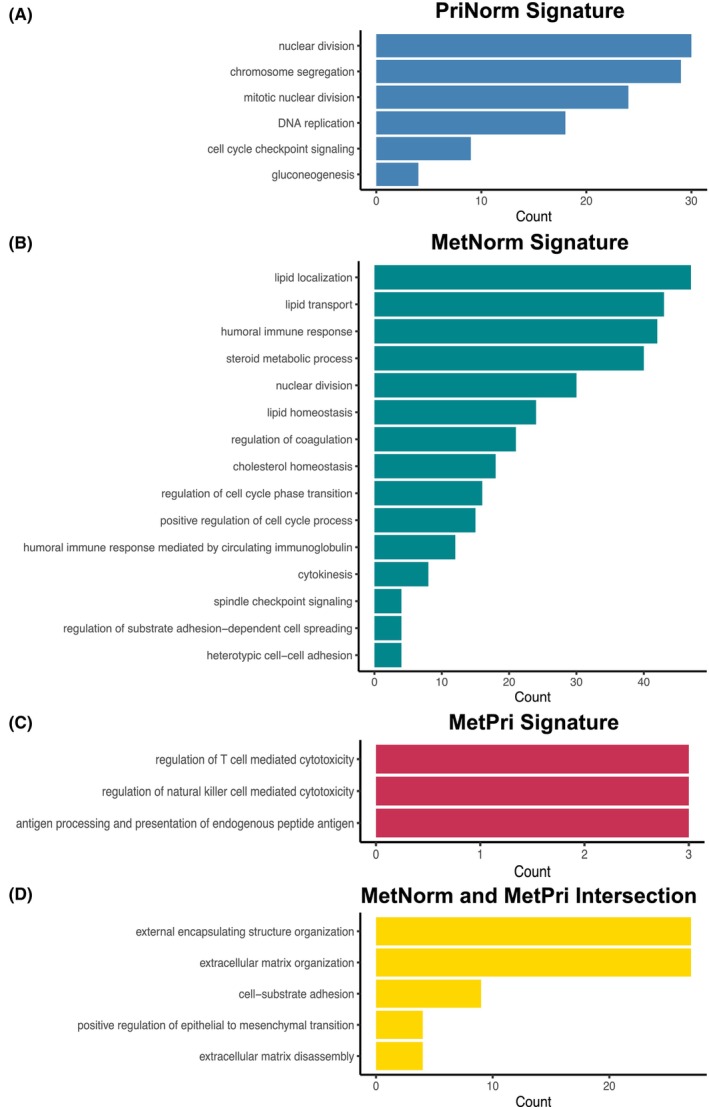
Gene Ontology enrichment (GO) of the genes regulated by exclusive master regulator (MRs). (A) GO of PriNorm signature. (B**)** GO of MetPri signature. (C**)** GO of MetNorm signature. (D**)** GO of MRs shared between MetNorm and MetPri signatures.

Figure 3 shows the functional enrichment of regulons exclusive of each gene signature. PriNorm‐exclusive regulons were associated with DNA replication, nuclear division, cell cycle regulation, and gluconeogenesis (Figure 3A and Table S2). MetNorm‐exclusive regulons were associated with lipid/steroid metabolism, immune response, nuclear division, and coagulation cascades (Figure 3B and Table S2). MetPri‐exclusive regulons were enriched to the regulation of cytotoxicity mediated by T lymphocyte and natural killer cells (NK), and presentation of peptide antigens (Figure 3C and Table S2). Regulons identified with both MetNorm and MetPri signatures were enriched to epithelial‐to‐mesenchymal transition, cell‐substrate adhesion, and extracellular matrix organization (Figure 3D and Table S2).

### 3.3 Symbolic regression

We applied symbolic regression modeling to identify the MRs with predictive capacity and potential as biomarkers in mCRPC. Ten models were generated for each combination of clinical outcomes (PriNorm: Primary CRPC vs. Normal Tissue, MetNorm: mCRPC vs. Normal Tissue, and MetPri: mCRPC vs. Primary CRPC) (Table S3).

We noted that HELLPAR was a recurrent feature in nearly all models in MetNorm and MetPri prediction, demonstrating high accuracy in predicting the metastatic group based on its expression (Table S3). According to the model, SNHG18 was the outstanding lncRNA in the PriNorm prediction, and its expression had the potential to identify the primary CRPC (Table S3). All the generated models showed AUC >0.95, demonstrating that the combination of previously MRs inferred successfully distinguished the clinical groups.

We re‐ran the symbolic regression using only HELLPAR and SNHG18 expressions since they drew attention based on previously modeled equations (Table 2). SNHG18 demonstrated high accuracy in predicting MetNorm and PriNorm, but not MetPri (Table 2). HELLPAR could not effectively discern the PriNorm. However, the addition of SNHG18 to the model improved the predictive performance of these groups (Table 2).

**TABLE 2:** Symbolic regression modeling with transcriptional expression of HELLPAR and SNHG18.

Model	AUC train	AUC test	Accuracy train	Accuracy test	Functional form (logreg())
PriNorm	1.0	0.99	1.0	0.95	SNHG18
MetNorm	1.0	0.99	0.96	0.96	SNHG18
MetPri	0.5	0.55	0.84	0.86	SNHG18
PriNorm	0.51	0.44	0.81	0.82	HELLPAR
MetNorm	1.0	1.0	1.0	1.0	HELLPAR
MetPri	1.0	1.0	1.0	1.0	HELLPAR
PriNorm	1.0	0.96	0.83	0.82	HELLPAR + SNHG18
MetNorm	1.0	1.0	1.0	0.98	HELLPAR + SNHG18
MetPri	1.0	1.0	0.97	1.0	HELLPAR + SNHG18

### 3.4 Master regulators (MRs) investigation

**FIGURE 4:**
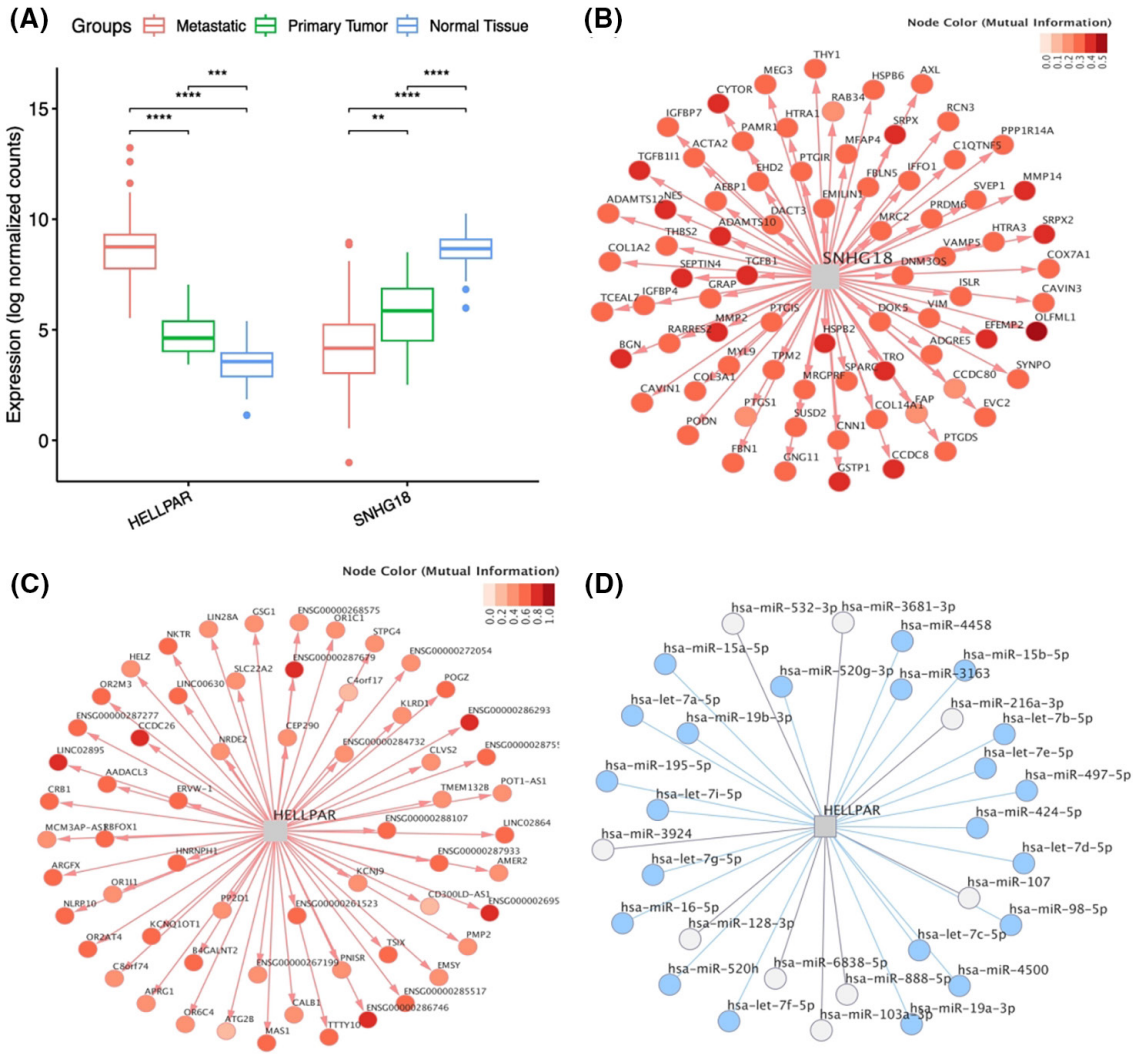
Gene expression profile and regulatory network of SNHG18 and HELLPAR. (A**)** Differential expression of HELLPAR and SNHG18 between three groups: metastatic CRPC, primary CRPC, and normal tissue. Statistics were performed by Mann–Whitney test with FDR correction. *p < 0.05, **p < 0.01, ***p < 0.001, ****p < 0.0001. (B) Coexpression network of SNHG18. (C) Coexpression network of HELLPAR. (D) ceRNA network of HELLPAR. Blue nodes indicate miRNAs that interact with HELLPAR and its target genes.

Figure 4A shows the expression of HELLPAR and SNHG18 in the mCRPC (obtained from TCGA—WCDT‐NCRPC) compared with primary prostate cancer (obtained from GSE80609) and normal prostate tissue (obtained from TCGA‐PRAD). HELLPAR was upregulated in the metastatic group compared to primary CRPC and normal tissue, while SNHG18 was downregulated (Figure 4A and Figure S4). Figure 4B,C demonstrate the target genes of these two lncRNAs. All HELLPAR and SNHG18 targets are positively regulated (red edge in Figure 4B,C), like most targets regulated by MetPri and MetNorm MRs (Figure S2).

We also investigated the activity of HELLPAR and SNHG18 as ceRNAs (competitive endogenous RNAs). Only HELLPAR had information in the prostate cancer cell line. Therefore, only their interactions were considered in ceRNAs' analysis. Figure 4D shows the interaction between HELLPAR and its putative target miRNAs. The blue nodes indicate miRNAs that also interact with HELLPAR targets, giving consistency to ceRNAs' hypothesis (Table S4). We believe that one of the mechanisms used by HELLPAR to regulate its targets is the competing endogenous network, where it competitively binding blue miRNAs and regulating mRNA expression.

## 4 DISCUSSION

Metastatic castration‐resistant prostate cancer (mCRPC) is an advanced form of cancer with a poor prognosis that remains incurable and deadly despite the advancements in therapies.^46^, ^47^ Understanding the molecular mechanism behind mCRPC is essential to elucidate the processes of castration resistance and metastatic spread and develop novel therapeutic strategies. It is well‐established that mCRPC development is driven by the androgen receptor (AR) signaling axis, directing several studies to understand how AR signaling is restored after androgen deprivation therapy (ADT).^48^ There are at least three hypotheses until now: (i) mCRPC develops independently of androgen, (ii) PCa cells acquire alterations in AR making it more sensitive to low androgen levels, or (iii) there is intratumoral androgen production. Overall, androgen triggers prostate tumor growth by altering the cell cycle and metabolic pathways, including lipid metabolism.^49^

Lipid metabolism is a hallmark of cancer that is activated in tumor cells to compensate for high metabolic demand, maintain cellular membranes, and act as a secondary messenger in cell signaling.^50^ In CRPC, lipid metabolism plays an additional role. Lounis et al.^51^ demonstrated that pathways associated with the synthesis of lipids are upregulated in the CRPC cell line compared to hormone‐sensitive prostate cancer (HSPC), playing an essential role in castration resistance. Furthermore, a combination of lipogenesis inhibitor (SCD1 INH) and AR antagonist (enzalutamide) was more effective in tumor regression than AR antagonist alone.^51^ Our results showed exclusive MetNorm regulons were enriched to lipid metabolism, cholesterol/sterol homeostasis, and cell cycle signaling, suggesting their role in cell proliferation and castration resistance in mCRPC. Two of these MRs (HAND2‐AS1 and LINC00261) are described in the literature associated with the maintenance of intracellular cholesterol^52^ and fatty acid metabolism,^53^ respectively. Several findings have supported the involvement of cholesterol in cell proliferation by maintaining membrane integrity and signal transduction.^54^ More recently, it has been proposed that cholesterol homeostasis is a precursor to de novo androgens synthesis, which triggers AR activation with consequent PCa progression. Accordingly, PCa cells regulate the enzymes involved in steroidogenesis and androgen catabolism to maintain testosterone levels within the tumor.^55^ Some of these enzymes, like CYP27A1,^56^ AKR1Cs,^57^ APOA‐1,^58^ and HSD17B2,^59^ are being regulated by MetNorm MRs in our results. These MRs may be essential in androgen metabolism, supporting the hypothesis of intratumoral androgen production by mCRPC.

SNHG18 and MEG3 were the unique MRs shared by MetNorm and MetPri signatures. MEG3 is a lncRNA downregulated in PCa that, when overexpressed, can inhibit cell proliferation, migration, and invasion.^60^ SNHG18 is a potential metastatic feature evidenced in several types of cancer, including hepatocellular carcinoma, lung cancer, and glioma.^61^, ^62^, ^63^ In our results, SNHG18 showed low expression in mCRPC and is regulating MMP‐2 and vimentin genes. MMP‐2 is an immunomodulator of cytoskeletal remodeling associated with tumor malignancy, invasiveness, and the ability to metastasize to distant organs that have been evidenced in prostate cancer for many years.^64^ Likewise, vimentin is a well‐established mesenchymal–epithelial transition marker detected in metastases‐derived prostate cancer cells.^65^ Based on this evidence, we suggest that SNHG18 is involved in mCRPC advancement by regulating several targets, especially MMP‐2 and vimentin.

Symbolic regression is a machine learning method to discover mathematical relationships between features and identify models that best fit a dataset. It has previously been applied to classify invasive and noninvasive forms of breast cancer, where it showed high accuracy.^42^ In the present study, we employed this approach as a complementary method to regulatory network analysis to find MRs combinations that lead to high predictive performance and identify potential biomarkers. We observed that the combination of MRs previously inferred by regulatory assessment effectively discriminates between the clinical groups, confirming their coordinated regulatory activity in mCRPC. SNHG18 appears in almost all models in PriNorm prediction. Its transcriptional expression efficiently distinguished mCRPC and primary CRPC from normal tissue. However, it fails to distinguish mCRPC from primary CRPC, suggesting a potential involvement in the metastatic cascade and castration resistance. More studies are needed to confirm this hypothesis.

HELLPAR was another highlight in symbolic regression modeling. It appears in most models constructed to distinguish metastasis from primary CRPC and normal tissue. Previous research has documented the high expression of this lncRNA in PCa and its role as a ceRNA (competing endogenous RNA) of miR‐30e in PCa cells. MiR‐30e targets several mRNAs, including the AR gene.^66^ Moreover, HELLPAR can interact with proteins involved in RNA splicing and ribosome roles, which are essential processes that drive cellular proliferation and tumorigenesis.^67^ In our results, the linear combination of HELLPAR and SNHG18 effectively discriminates between the clinical groups, suggesting that they act together in the mCRPC development.

The lncRNAs are promising targets to therapy and prognosis due to their detectability in tissues and body fluids, specific expression in particular cells, and susceptibility to low‐dose drugs, thereby minimizing the toxicity commonly associated with therapies.^16^ They acting in gene regulation through (i) protein recruitment involved in chromatin epigenetic modification, mRNA splicing, mRNA transcription, and signaling pathways, (ii) generating hybrid structure with DNA and influencing accessibility to it, (iii) pairing with other RNAs involved in mRNA regulation, (iv) acting as scaffolds for proteins, and (v) acting as sponges for miRNAs (also known as ceRNAs).^68^ In the present study, we investigated the activity of HELLPAR as ceRNA and identified 31 miRNAs that are regulated by it. Notably, 22 of these miRNAs were found to also interact with HELLPAR targets. So, we hypothesized that these 22 miRNAs act on the competitive endogenous network in mCRPC. Some of these miRNAs have been previously documented in the literature to be associated with PCa, including has‐miR‐19a‐3p, hsa‐miR‐19b‐3p, miR‐16‐5p, hsa‐miR‐195‐5p, hsa‐miR‐497‐5p, hsa‐let‐7 g‐5p, hsa‐let‐7, and hsa‐miR‐98‐5p.^69^, ^70^, ^71^, ^72^, ^73^, ^74^, ^75^, ^76^ The let‐7 miRNAs, specifically, is a family of miRNAs that act as tumor suppressors in CRPC cells.^69^

In general, the lncRNAs can bind to multiple miRNAs, as well as genes can be regulated by the coordinated activity of two or more lncRNAs, creating a complex regulatory network.^68^ In this study, we demonstrated the interaction between lncRNA‐mRNAs and demonstrated a possible mechanism by which this regulation occurs—miRNA sponge. However, in vitro experiments are needed to validate the functions of SNHG18 and HELLPAR.

## 5 CONCLUSIONS

The current findings contribute to understanding the regulatory behavior of lncRNA in mCRPC and support the involvement of lipid metabolism and androgen signaling in this tumor. Although there may be other pathways involved in this tumor type, the androgen role in mCRPC is undeniable. We also evidenced SNHG18 and HELLPAR as central regulators in the mCRPC transcriptional network and possible diagnostic targets. Further exploration is required to understand how the lncRNA target gene regulation occurs in mCRPC. It may involve either competitive endogenous mechanisms or the scaffold of the RNA‐protein complex. In this study, we investigated all transcriptomic mCRPC samples available public database. However, In vitro assays seem to be the natural next step to better investigate SNHG18 and HELLPAR functionalities, as well as the impact of SNHG18 and HELLPAR in patients' overall survival. Moreover, it is necessary to validate these molecules in cellular and animal models to enable their application in therapeutic or diagnostic approaches.

## AUTHOR CONTRIBUTIONS

**Rafaella Sousa Ferraz:** Conceptualization (equal); data curation (equal); writing – original draft (equal); writing – review and editing (equal). **João Vitor Ferreira Cavalcante:** Conceptualization (equal); data curation (equal); formal analysis (equal). **Leandro Magalhães:** Writing – review and editing (equal). **Andrea Ribeiro dos Santos:** Conceptualization (equal); funding acquisition (equal); writing – review and editing (equal). **Rodrigo Juliani Siqueira Dalmolin:** Conceptualization (equal); funding acquisition (equal); project administration (equal); supervision (equal); writing – original draft (equal); writing – review and editing (equal).

## CONFLICT OF INTEREST STATEMENT

The authors declare no conflict of interests.

## Supporting information


Figures S1–S4

Table S1

Table S2

Table S3

Table S4

## Data Availability

The datasets analyzed during the current study are available in Gene Expression Omnibus (GEO) with accession numbers GSE126078 and GSE80609, and in The Cancer Genome Atlas (TCGA) with collection name TCGA‐PRAD and WCDT‐MCRPC. The source code can be found at this repository: https://github.com/dalmolingroup/Network‐lncRNA‐mCRPC
