# Supplementary material for: Revealing metastatic castration‐resistant prostate cancer master regulator through lncRNAs‐centered regulatory network
Source: Cancer Med. 2023 Aug 29;12(18):19279–90. doi: 10.1002/cam4.6481 (PMC10557827; doi:10.1002/cam4.6481)
Supplement: Supplementary file 1 — Figures S1–S4 [file CAM4-12-19279-s002.pdf]

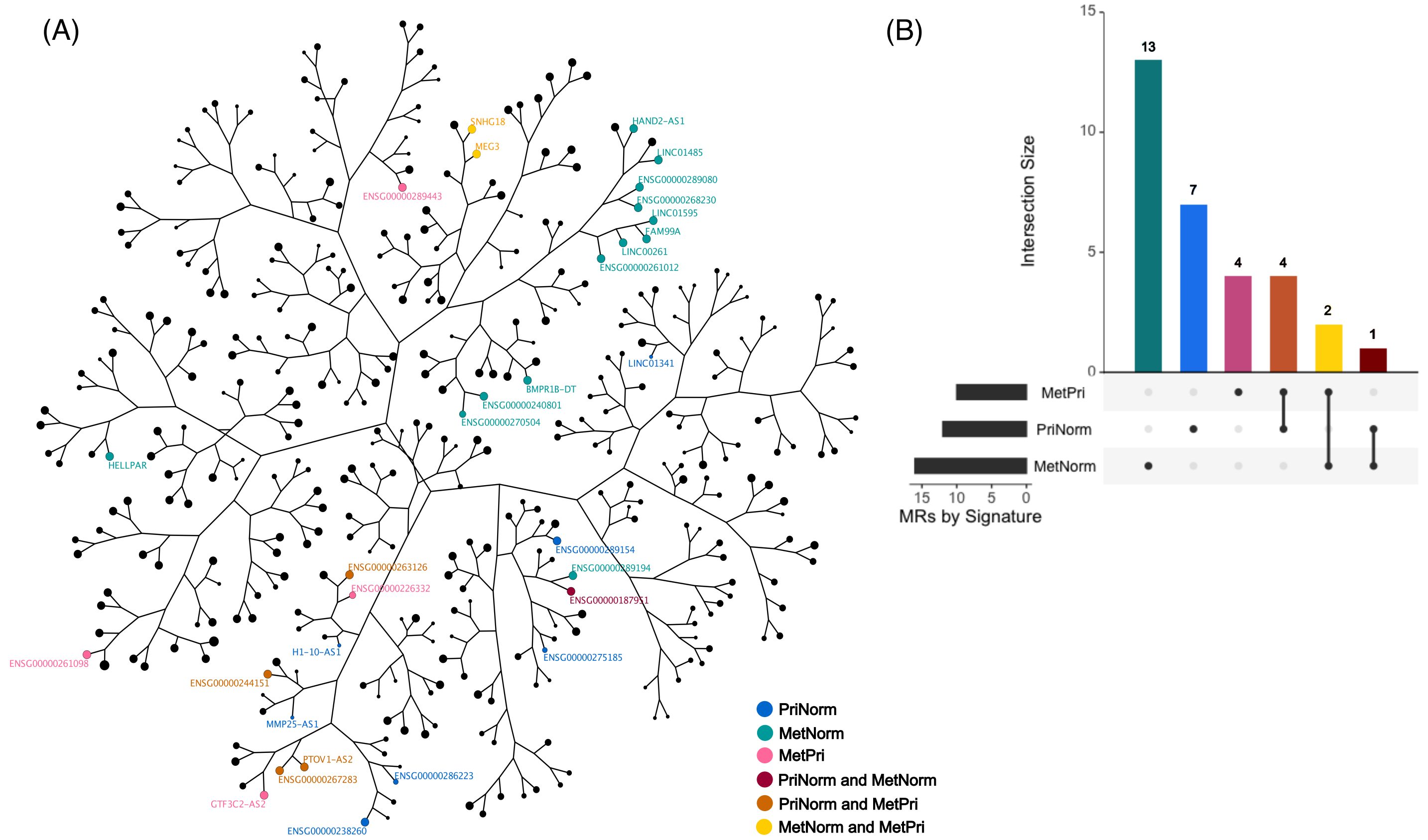

Supplementary Figure 1 Metastatic Castration-Resistant Prostate Cancer (mCRPC) network. **A** Tree-and-leaf representation of mCRPC network. Nodes represent regulons labeled by their lncRNA and colored according to Master Regulator (MRs) Analysis. **B** Upset plot indicating the number of MRs shared between gene signatures.

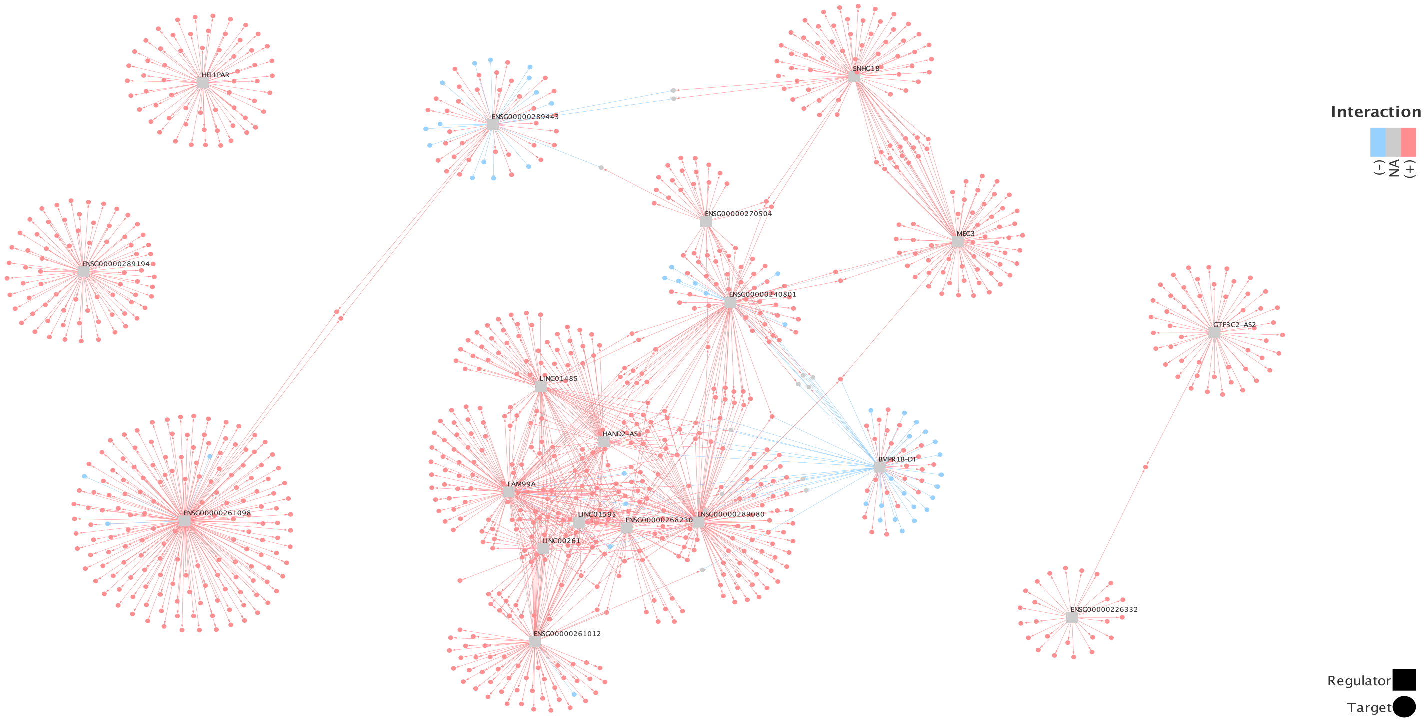

Supplementary Figure 2 Small portion of the mCRCP network featuring Master Regulator (MRs) identified in the MetPri and MetNorm signatures. Blue circle represents genes with a negative association, red circle represents genes with positive association, and gray circle represents genes that receive antagonistic regulation. Gray squared represents regulons labeled by their lncRNA.

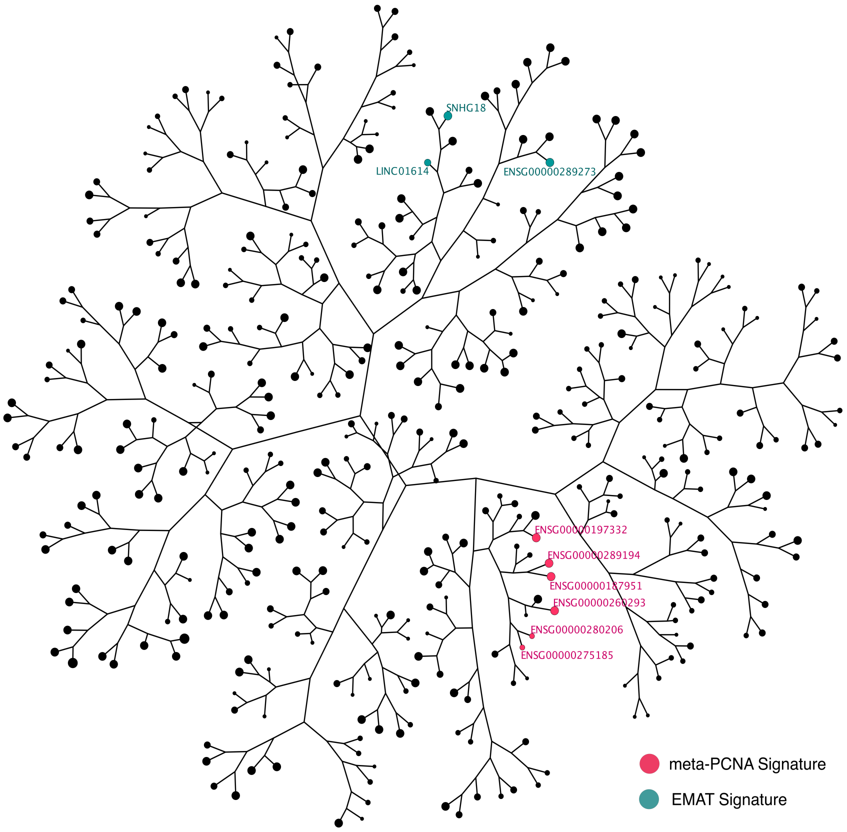

Supplementary Figure 3 Tree-and-leaf representation of mCRPC network with nodes colored according to EMAT and PCNA signatures.

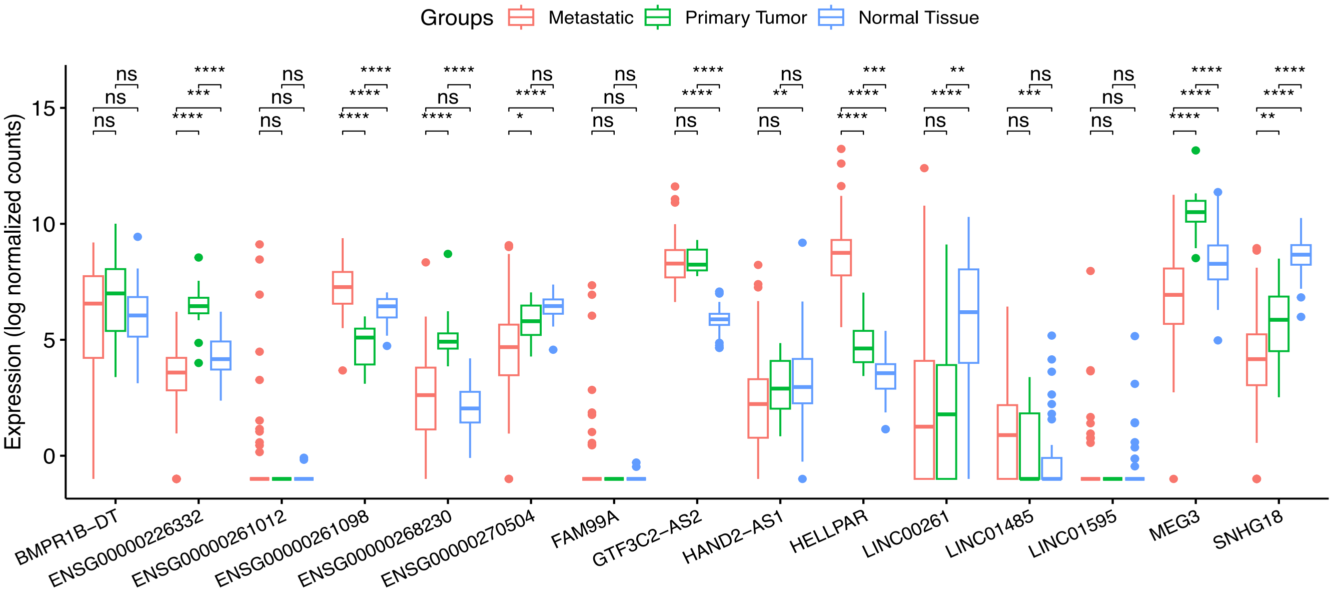

Supplementary Figure 4 Differential expression analysis of exclusive Master Regulator (MRs) from MetNorm and MetPri signatures. Statistics were performed by Mann-Whitney test with FDR correction. *p < 0.05, **p < 0.01, ***p < 0.001, **** p < 0.0001.
