# Supplementary material for: Revealing metastatic castration‐resistant prostate cancer master regulator through lncRNAs‐centered regulatory network
Source: Cancer Med. 2023 Aug 29;12(18):19279–90. doi: 10.1002/cam4.6481 (PMC10557827; doi:10.1002/cam4.6481)
Supplement: Supplementary file 2 — Table S1 [file CAM4-12-19279-s001.docx]

**Supplementary Table 1.** Table of lncRNAs regulated by exclusive Master Regulator (MRs) from MetNorm and MetPri signatures

| N | Regulon | Targets (lncRNAs) |
| --- | --- | --- |
| 1 | SNHG18 | MEG3,CYTOR,DNM3OS |
| 2 | MEG3 | MEG9,MIR503HG,ENSG00000240801,SNHG18 |
| 3 | FAM99A | LINC01595,ENSG00000261012,ENSG00000268230,ENSG00000289080 |
| 4 | ENSG00000289080 | FAM99A,LINC01554 |
| 5 | ENSG00000261012 | FAM99A,LINC02532,LINC01485,LINC01348 |
| 6 | HELLPAR | CD300LD-AS1,APRG1,MCM3AP-AS1,LINC00630,POT1-AS1,CCDC26,TTTY10,LINC02895,ENSG00000261523,LINC02864,  ENSG00000267199,ENSG00000268575,ENSG00000269514,KCNQ1OT1,TSIX,ENSG00000272054,ENSG00000285517,ENSG00000286293,ENSG0000028674,ENSG00000287277,ENSG00000287558,ENSG00000287679,ENSG0000028793,ENSG00000288107 |
| 7 | HAND2-AS1 | DBH-AS1,LINC01485 |
| 8 | LINC01485 | HAND2-AS1,ENSG00000261012,ENSG00000286733 |
| 9 | LINC01595 | FAM99A |
| 10 | ENSG00000289194 | ENSG00000259865 |
| 11 | BMPR1B-DT | ENSG00000233516,ARLNC1,ENSG00000263427 |
| 12 | ENSG00000240801 | H19,MEG3,DIO3OS |
| 13 | ENSG00000268230 | FAM99A,LINC01554 |
| 14 | ENSG00000289443 | ENSG00000228113,ANKRD10-IT1,LINC01198,ENSG00000232909,MIRLET7IHG,ENSG00000261642,ENSG00000289688 |
| 15 | GTF3C2-AS2 | ENSG00000232098,ENSG00000238260,ENSG00000244151,ENSG00000257900,ENSG00000266340,ENSG00000289154 |
| 16 | ENSG00000261098 | ENSG00000233928,NFYC-AS1 |
| 17 | ENSG00000226332 | ENSG00000260279,ENSG00000267283 |
