# Supplementary material for: Revealing metastatic castration‐resistant prostate cancer master regulator through lncRNAs‐centered regulatory network
Source: Cancer Med. 2023 Aug 29;12(18):19279–90. doi: 10.1002/cam4.6481 (PMC10557827; doi:10.1002/cam4.6481)
Supplement: Supplementary file 4 — Table S3 [file CAM4-12-19279-s005.docx]

**Supplementary Table 3.** Symbolic regression models that best predict the clinical groups: Metastatic, Primary CRPC, and Normal Tissue.

| Model | AUC  Train | AUC  Test | Accuracy  Train | Accuracy  Test | BIC | N. Features | Functional form (logreg()) | Loss |
| --- | --- | --- | --- | --- | --- | --- | --- | --- |
| PriNorm | | | | | | | | |
| 1 | 1.0 | 0.99 | 1.0 | 0.95 | 0.0 | 1 | SNHG18 | 2.65e-05 |
| 2 | 1.0 | 1.0 | 1.0 | 1.0 | 3.74 | 2 | ENSG00000263126 + SNHG18 | 5.22e-06 |
| 3 | 1.0 | 1.0 | 1.0 | 1.0 | 3.74 | 2 | ENSG00000268230 + SNHG18 | 5.37e-06 |
| 4 | 1.0 | 1.0 | 1.0 | 0.95 | 3.74 | 2 | ENSG00000244151 + SNHG18 | 5.77e-06 |
| 5 | 1.0 | 1.0 | 1.0 | 0.95 | 3.74 | 2 | ENSG00000238260 + SNHG18 | 6.18e-06 |
| 6 | 1.0 | 1.0 | 1.0 | 1.0 | 3.74 | 2 | ENSG00000261098 + ENSG00000263126 | 6.66e-06 |
| 7 | 1.0 | 1.0 | 1.0 | 1.0 | 3.74 | 2 | ENSG00000261098 + SNHG18 | 7.26e-06 |
| 8 | 1.0 | 1.0 | 1.0 | 0.91 | 3.74 | 2 | LINC01485 + SNHG18 | 7.47e-06 |
| 9 | 1.0 | 1.0 | 1.0 | 0.95 | 3.74 | 2 | LINC00261 + SNHG18 | 7.62e-06 |
| 10 | 1.0 | 0.97 | 1.0 | 0.95 | 3.74 | 2 | ENSG00000268230 + PTOV1-AS2 | 7.66e-06 |
| MetNorm | | | | | | | | |
| 1 | 1.0 | 1.0 | 1.0 | 1.0 | 0.09 | 1 | HELLPAR | 0.000429 |
| 2 | 1.0 | 0.99 | 1.0 | 0.96 | 4.62 | 2 | GTF3C2-AS2 + SNHG18 | 6.49e-06 |
| 3 | 1.0 | 1.0 | 1.0 | 0.96 | 4.62 | 2 | GTF3C2-AS2 + HELLPAR | 8.38e-06 |
| 4 | 1.0 | 1.0 | 1.0 | 1.0 | 4.62 | 1 | 1/HELLPAR | 1.26e-05 |
| 5 | 1.0 | 1.0 | 1.0 | 0.98 | 4.62 | 2 | HELLPAR + SNHG18 | 1.94e-05 |
| 6 | 1.0 | 1.0 | 1.0 | 1.0 | 4.64 | 1 | 2*HELLPAR | 0.000104 |
| 7 | 1.0 | 1.0 | 1.0 | 0.98 | 4.64 | 2 | HELLPAR + LINC01341 | 0.000118 |
| 8 | 1.0 | 1.0 | 1.0 | 0.98 | 4.64 | 2 | ENSG00000268230 + HELLPAR | 0.000119 |
| 9 | 1.0 | 1.0 | 1.0 | 1.0 | 4.65 | 2 | ENSG00000226332 + HELLPAR | 0.000192 |
| 10 | 1.0 | 0.98 | 1.0 | 0.94 | 4.66 | 2 | ENSG00000187951 + SNHG18 | 0.000235 |
| MetPri | | | | | | | | |
| 1 | 1.0 | 1.0 | 1.0 | 1.0 | 0.2 | 1 | HELLPAR | 0.001362 |
| 2 | 1.0 | 1.0 | 1.0 | 1.0 | 4.31 | 2 | ENSG00000238260 + ENSG00000261098 | 9.70e-06 |
| 3 | 1.0 | 1.0 | 1.0 | 1.0 | 4.31 | 2 | ENSG00000238260 + HELLPAR | 1.01e-05 |
| 4 | 1.0 | 1.0 | 1.0 | 1.0 | 4.31 | 2 | HELLPAR + LINC01341 | 1.03e-05 |
| 5 | 1.0 | 1.0 | 1.0 | 1.0 | 4.31 | 2 | HELLPAR + PTOV1-AS2 | 1.08e-05 |
| 6 | 1.0 | 1.0 | 1.0 | 1.0 | 4.31 | 2 | ENSG00000261098 + PTOV1-AS2 | 1.09e-05 |
| 7 | 1.0 | 1.0 | 1.0 | 1.0 | 4.31 | 2 | H1-10-AS1 + HELLPAR | 1.48e-05 |
| 8 | 1.0 | 1.0 | 1.0 | 1.0 | 4.31 | 2 | ENSG00000263126 + HELLPAR | 1.56e-05 |
| 9 | 1.0 | 0.97 | 1.0 | 0.97 | 4.31 | 2 | ENSG00000268230 + HELLPAR | 1.58e-05 |
| 10 | 1.0 | 1.0 | 1.0 | 1.0 | 4.31 | 2 | ENSG00000244151 + HELLPAR | 1.88e-05 |
